# Early psychosocial parent–infant interventions and parent–infant relationships after preterm birth–a scoping review

**DOI:** 10.3389/fpsyg.2024.1380826

**Published:** 2024-08-07

**Authors:** Marika Leppänen, Riikka Korja, Päivi Rautava, Sari Ahlqvist-Björkroth

**Affiliations:** ^1^Department of Psychiatry and Public Health, University of Turku and Turku University Hospital, Turku, Finland; ^2^Department of Psychology, University of Turku, Turku, Finland; ^3^Department of Public Health, University of Turku, and Research Services, Turku University Hospital, Turku, Finland; ^4^Department of Clinical Medicine, Turku University Hospital, Turku, Finland

**Keywords:** preterm born infant, psychosocial intervention, parent-infant relationship, parent-infant interaction, parenting a child with disabilities

## Abstract

**Objective:**

Early psychosocial interventions for preterm infants and their parents are diverse. This study aimed to structure the knowledge on psychosocial parent–infant interventions and to identify gaps in the intervention studies.

**Methods:**

We included studies on early (during first year of life) psychosocial parent–infant interventions with parent–infant relationship outcomes after preterm birth (< 37 weeks). We excluded studies that did not focus on preterm infants, failed to indicate the studied intervention and outcomes, were not written in English, were not controlled or peer-reviewed studies, or did not provide essential information for eligibility. The search included studies published between January 2000 and March 2024 in PubMed and PsycINFO. Preferred Reporting Items for Systematic Reviews and Meta-Analyses (PRISMA) guidelines were followed in reporting. Psychosocial parent–infant intervention studies were classified adapting the International Classification of Health Interventions (ICHI) and the Template for Intervention Description and Replication (TIDieR).

**Results:**

The included 22 studies reported data from 18 different interventions with preterm infants (< 37 weeks). Studies excluded preterm infants with health risks (19/22, 86%), with very low gestational age and/or birth weight (7/22, 32%), and/or mothers with psychosocial risks (14/22, 64%). Of the 18 interventions, 12 (67%) were classified as counseling, 3 (17%) as emotional support, 2 (11%) as psychotherapeutic, and 1 (6%) as educational. The parent–child relationship was assessed using 30 different methods and varying time points up to 18 months of age. Most studies (17/22, 77%) reported positive changes in the parent–child relationship favoring the intervention group.

**Conclusion:**

We identified four types of interventions to influence parenting behavior; the most used was counseling. All four intervention types showed positive effects on parent–infant relationships, although the preterm populations studied were selective, the effects were evaluated using different methods, and the follow-up periods were short. These findings indicate a need for studies with standardized methods, longer follow-up, and less-restricted preterm populations to develop guidelines for all families with preterm infants.

## Introduction

Preterm birth (< 37 weeks of gestation) is a stressor for infant brain development (Krugers et al., 2017) and may relate to adverse neurodevelopmental (Bhutta et al., 2002; Aarnoudse-Moens et al., 2013) and socioemotional outcomes in later life (Bhutta et al., 2002; Aarnoudse-Moens et al., 2009; Woodward et al., 2009; Burnett et al., 2011; D’Onofrio et al., 2013; Johnson and Marlow, 2013). The lower the gestational age of the infant at birth, the higher the risk for developmental problems (Elgen et al., 2012; Arpi and Ferrari, 2013), hospital readmissions, and special care (Clark et al., 2008; Larroque et al., 2008; Athalye-Jape et al., 2022). Therefore, children born preterm are fragile and need a good environment to overcome early challenges and develop well.

A preterm birth is also a challenge to transition to parenthood. It interrupts the psychological preparation for the birth, leads often to early separation from the infant, and can include psychologically stressful or even traumatic situations during labor or the neonatal intensive care unit (NICU) care of the infant (Miles and Holditch-Davis, 1997; Jotzo and Poets, 2005; Melnyk et al., 2006; Forcada-Guex et al., 2011; Baum et al., 2012; Yaari et al., 2019). Earlier studies have shown that preterm birth also relates to stress and elevated mental health symptoms in parents, which, in turn, may negatively affect the parent–infant relationship (Forcada-Guex et al., 2011; Meijssen et al., 2011; Shah et al., 2011; Korja et al., 2012; Gerstein and Poehlmann-Tynan, 2015; Hoffenkamp et al., 2015a; Ionio et al., 2017; Gerstein et al., 2019). Together, infant fragility and challenges to early parenthood can create a complex negative circle of prematurity.

A well-functioning family relationships are a protective factor for the development of a fragile preterm infant (Miceli, 2000; Gross et al., 2001; Treyvaud et al., 2012; Aarnoudse-Moens et al., 2013; Gerstein and Poehlmann-Tynan, 2015; Faure et al., 2017). The optimal time to intervene in family relationships is during early months of an infant’s life and parenthood, as this is a sensitive period in infant development and the biopsychological processes of parenthood (Givrad et al., 2021). Therefore, the interventions supporting parenting and the parent–infant relationships (Muller-Nix and Forcada-Guex, 2009; Zeanah, 2009; Meijssen et al., 2011; Benzies et al., 2013; Gerstein and Poehlmann-Tynan, 2015; Givrad et al., 2021) should be important part of modern neonatal care.

There is evidence available that interventions supporting parenting or specifically emotional parent–infant relationships during NICU stay and/or the early months after discharge lead to positive health outcomes (Benzies et al., 2013; Evans et al., 2014; Yrjölä et al., 2022). In particular, interventions that include direct support for the parents or focus on the parent–infant interaction have been shown to improve outcomes such as parents’ mental well-being, child rearing attitudes, and the socioemotional and cognitive development of prematurely born children (Nordhov et al., 2010; Landsem et al., 2014, 2020; Welch et al., 2015; Vohr et al., 2017). A meta-analytic review showed that 8 out of 17 different psychosocial parent–infant interventions that were tested in randomization-controlled trials and published up to 2007 were effective in improving the quality of the parent–infant relationship. These eight interventions (Mother Infant Transaction Program, State Modulation, Nursing Systems Toward Effective Parenting-Preterm, Infant Behavioral Assessment and Intervention Program, Guided Participation, Kangaroo Holding, Traditional Holding, and an individualized family-based intervention) focused mainly on cue-based and responsive parental care (Evans et al., 2014).

However, a recent systematic review of early interventions for parenting in NICU found only small and short-term effects on parental sensitivity and stress compared with usual care or basic educational guidance, possibly because of diversity, implementation failure, or methodological bias (Lavallée et al., 2021). The structural framework of psychosocial parent–infant interventions in the preterm context has also been shown to be very heterogeneous, leaving uncertainties about their effectiveness and understanding of what works for whom (Benzies et al., 2013; Cho et al., 2013; Evans et al., 2014; Puthussery et al., 2018; Givrad et al., 2021; Lavallée et al., 2021). There have been no attempts to address this heterogeneity of intervention studies, for example, whether there are different types of interventions, and if fathers or different family structures were taken into consideration. There may be different subgroups of parents and infants and thereby a need for different approaches. For example, children born very preterm are at risk for different mental and behavioral disorders than children born preterm with later gestational age (Leppänen et al., 2023) and even for out-of-home placement during early years (Alenius et al., 2020). In the group of very preterm infants maternal psychosocial factors play an important role in the actualization or mitigation of these risks (Leppänen et al., 2023). Identifying appropriate interventions for different risk groups among NICU infants and families would be important.

A detailed understanding of the structural frameworks of the interventions and the level of support they provide would be especially beneficial for the planning of health care services (Castelpietra et al., 2017). However, no model has been designed for comparing and analyzing the differences between psychosocial parent–infant interventions for preterm infants and parents. Therefore, the aim of this scoping review was to provide more structured knowledge of early psychosocial parent–infant interventions that aim to promote parent–infant relationships during the first year after preterm birth. Specifically, we intended to explore the content of the interventions according to the theories, aims, implementation, and settings of the interventions and to study the parent–infant relationship outcomes. As described earlier, psychosocial parent–infant interventions vary a lot structurally; thus, we aimed to analyze the psychosocial parent–infant interventions in detail by using classification systems to identify potential different intervention types and describe parent–infant relationship outcomes. Furthermore, we aimed to summarize the evidence and identify potential gaps in the interventions and their implementation to benefit future research and health care services for parents with preterm infants.

## Methods

In this scoping review, the systematic search and reporting followed the Preferred Reporting Items for Systematic Reviews and Meta-Analyses (PRISMA) guidelines for scoping reviews (Page et al., 2021). The literature search was conducted for articles published from January 2000 to March 2020 and later for articles published April 2020 to March 2024.

### Eligibility criteria

We included studies describing early (between birth and first year) psychosocial parent–infant (psychotherapy and parental) interventions for parent(s) and preterm-born infants (< 37 weeks of gestation) with studied outcomes in the parent–infant relationship and which were written in English. We excluded studies if the infants were not preterm (i.e., were born >37 weeks), did not trial interventions for parents and infants, or did not provide parent–infant relationship outcomes.

### Search strategy

We reviewed the literature from 2000 to 2024 to examine the psychosocial parent–infant interventions and the parent–infant relationship outcomes of children born preterm in the 21st century, when neonatal care has reached an advanced stage. As earlier reviews did not search for psychotherapy/psychotherapeutic interventions, we included these as well. Only publications in English were included. We excluded studies that did not follow randomization control or study-control settings or essential information about the intervention to assess eligibility. Because we wanted to focus clearly on psychosocial interventions, we excluded interventions that promoted only physical closeness or one aspect of care, such as skin-to-skin contact. We also removed duplicates.

### Screening the data

**Figure 1:**
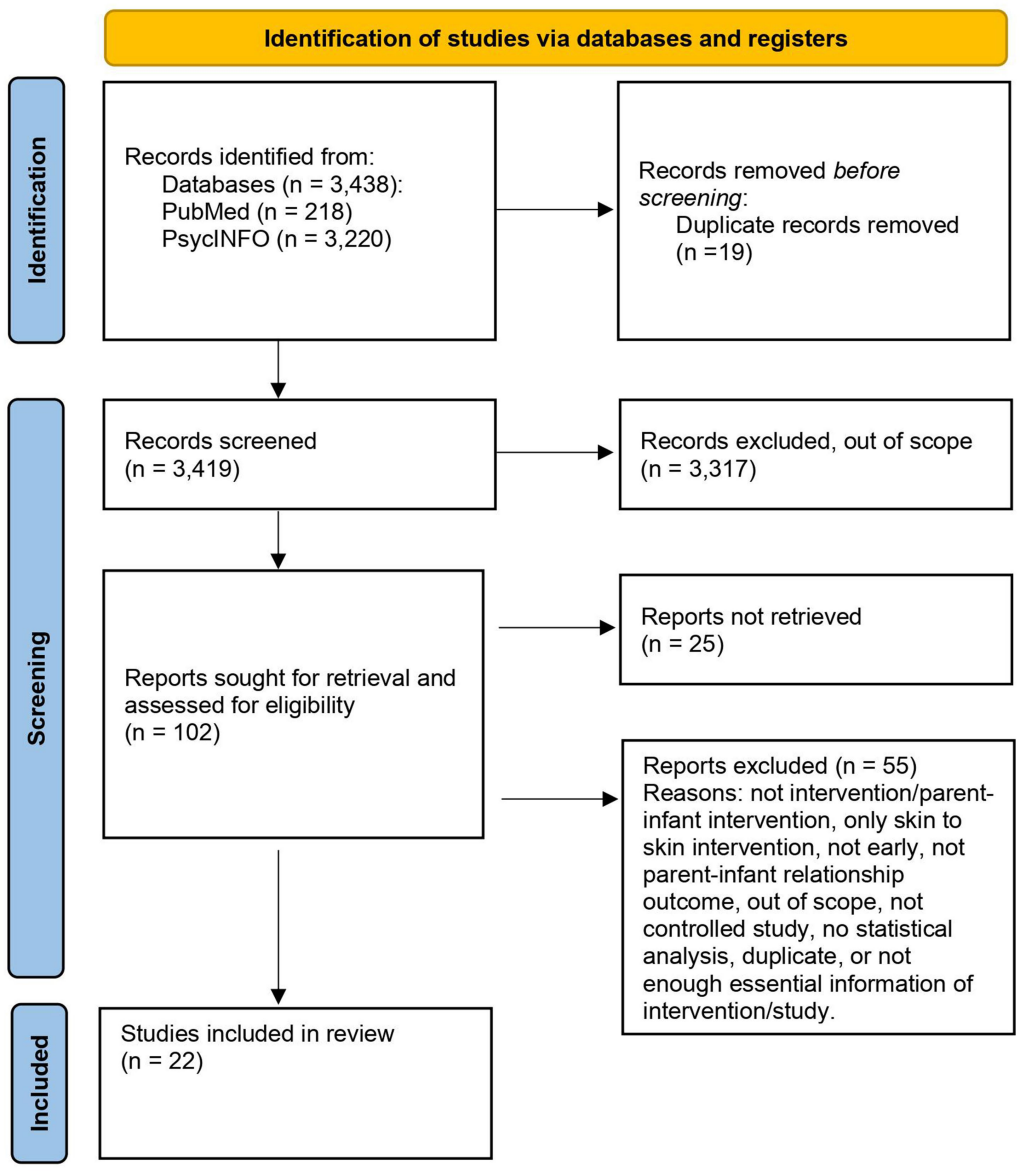
Flow diagram of included studies.

We searched the electronic databases PubMed and PsycINFO for all published data from January 2000 to March 2020. The search strategy comprised the following MESH headings or keywords: Premature Birth, Infant, Psychotherapy, Parent Infant Psychotherapy, and Parent Intervention. Searches were done in PubMed using the following criteria: “Premature Birth/psychology” [Mesh] OR “Infant, Premature/psychology” [Mesh] AND “Psychotherapy” [Mesh] OR “Parent Infant psychotherapy” [All Fields] OR “Parent Intervention” [All Fields] AND ((“2000/01/01” [PDAT]: “2020/03/01” [PDAT]) AND English[lang]). In PsycINFO, we used “Premature Birth” OR “Infant, Premature” AND “Psychotherapy” OR “Parent Infant psychotherapy” OR “Parent Intervention” Published Date: 20000101–20,200,331; Peer Reviewed; Publication Type: Peer Reviewed Journal; English; Age Groups: Childhood (birth−12 years); Population Group: Human; and Document Type: Journal Article. The number of all included and excluded articles of the literature search is provided in Figure 1, built according to the PRISMA guidelines (2021). Because reporting of the results took a long time, a complementary and identical search was done for all published data from April 2020 to March 2024. Studies were also identified from the reference lists of the screened manuscripts. The first author screened potential studies (*n* = 2,770) using the content analysis of the study abstracts and screened for eligibility by reading the manuscripts (*n* = 111). There were 8 studies identified through citations and were screened for eligibility. After screening, potential studies were scrutinized in greater depth through the reading of 74 manuscripts by the first author, with help of the coauthors, to examine the study methods, participants, inclusion/exclusion criteria, interventions (theory, aim, and implementation), outcomes, and results to conclude whether each study was eligible. In another search, the first author screened potential studies (*n* = 641) and scrutinized 21 of these studies, 4 of which were assessed in detail for eligibility with the help of the coauthors.

### Data extraction

For different types of early psychosocial parent–infant interventions, we chose four index terms from the International Classification of Health Interventions (ICHI): Parents in Health Intervention.^1^ The four main types chosen were: (1) “Education to influence parenting behavior”—Providing information to improve knowledge and influence behavior concerning patterns of interaction between a parent and their child/children, including the nature and degree of monitoring and supervision; involvement and engagement; discipline; nurturing; and the expression of affection. (2) “Counseling about parenting behavior”—Providing therapeutic and/or supportive communication in relation to behavior concerning patterns of interaction between a parent and their child/children, including the nature and degree of monitoring and supervision; involvement and engagement; discipline; nurturing; and the expression of affection. (3) “Emotional support for parenting behaviors”—Providing comfort, empathy, or motivational support to the person regarding behavior concerning patterns of interaction between a parent and their child/children, including the nature and degree of monitoring and supervision; involvement and engagement; discipline; nurturing; and the expression of affection. (4) “Psychotherapy for parenting behaviors”—providing therapeutic communication, based upon the systematic application of psychological theory, in relation to behavior concerning patterns of interaction between a parent and their child/children, including the nature and degree of monitoring and supervision; involvement and engagement; discipline; nurturing; and the expression of affection. In this manuscript, we use abbreviated versions of the models’ names: education, counseling, emotional support, and psychotherapy.

To categorize the interventions into the four different intervention types, we looked at the aims and theoretical backgrounds of the interventions but especially the implementation in terms of what was done, how it was done and who did it. In addition, whether there was a focus on reading infants’ cues or providing emotional support to parents or on the parent–infant relationship or psychotherapeutic approach. The ICHI definitions were applied to the practice with preterm infants (see results and discussion for examples).

All authors of this paper studied the psychosocial parent–infant interventions separately and used a common Microsoft Excel model to capture each intervention’s theory and content. A consensus (a minimum of two reviewers) was then reached to assign each study to one of the four intervention categories.

However, there were still details in the implementation of the interventions that separated the 18 individual interventions from each other, and we added the subtype classification to structure this information. This classification is shown in Table 1, which presents the four main types and the six subtypes. The first author used the Template for Intervention Description and Replication (TIDieR) checklist (Hoffmann et al., 2014) to structure the studied interventions using more detailed qualitative and structural information. The TIDieR checklist’s details, such as name, why (theory, aim), what (materials/counseling, general/individualized), who (intervention provider’s education/training), and how (implementation, e.g., online/face-to-face), as well as where, when, and how much (environment, frequency) and how well the planned compared to the actual work done were described, were used for subtype classification. We also described the parent–infant relationship outcomes of the studies, and in relation to early parental psychosocial parent–infant intervention types. Data are available on request.

**Table 1:** Classification of early psychosocial parent–infant interventions to influence parenting behaviors.

Education	Counseling	Emotional support	Psychotherapy
Subtype 1	Subtype 1 (or more)	Subtype 4	Subtype 5
*Creating Opportunities for Parent Empowerment (COPE)* (Melnyk et al., 2006, 2008)	*Family Based Intervention* (Browne and Talmi, 2005)* Baby Triple P (BTP) (Evans et al., 2017)**	*Modifications of MITP* (Kaaresen et al., 2006; Olafsen et al., 2008; Nordhov et al., 2010) *Early Intervention (EI)* (Borghini et al., 2014)	*Triadic Parent–Infant Relationship Therapy(TRT)* (Castel et al., 2016)
	Subtype 2	Subtype 5	Subtype 6
	*Guided Participation (GP)*(Schroeder and Pridham, 2006) *Mother–Infant Transaction Program (MITP)* (Ravn et al., 2012) *Mindfulness-Based Neurodevelopmental Care* (Petteys and Adoumie, 2018) *Newborn Individualized Developmental Care and Assessment Program (NIDCAP)* (van der Pal et al., 2008) *Family Nurturing Intervention (FNI)* (Beebe et al., 2018)	*Japanese Infant Mental Health Program (JIMPH)* (Cho et al., 2013)	*Early Preventive Attachment Oriented Psychotherapeutic* (Brisch et al., 2003)
	Subtype 3		
	*Parent Baby Interaction Program (PBIP)* (Glazebrook et al., 2007) *Infant Behavioral Assessment and Intervention Program (IBAIP)* (Meijssen et al., 2010, 2011) *Modification of Video Interaction Guidance (VIG)* (Hoffenkamp et al., 2015b) *Modifications of MITP* (Newnham et al., 2009)***		
	Subtype unknown		
	*Parent Training in Music and Multimodal Stimulation* (Whipple, 2000)****		

*Two different intervention groups, one without in-person contact; **Only 67% received the full protocol, for instance 11% received only audio material and phone calls; ***The number of intervention sessions unclear, 7 + 2 in protocol, but the order and pace of the content were adjusted to mothers’ needs; ****provider was unclear. Unknown, If there was missing relevant information about the intervention (dose, provider, other). Subtype 1, Only education material, no in-person contact, with very few contacts (1–4). Subtype 2, Short in-person support (1–4 sessions), counseling might notice infant’s individuality, and provider could take individual account of caretaking of this infant. A provider could be a health care worker with or without any specific training/supervision. Subtype 3, Longer counseling (> 5 sessions) and could last after discharge or shorter intervention with a specific tool (less than 6 months), provider had special training in program that aimed to enhance parent–infant relationship, the provider had supervision available, and intervention aimed to support parents to find infant’s unique characteristics, cues, and how parents could respond to and support infant better. Subtype 4, Similar to subtype 3, but also included possibility for more intense, but maximum 6 months, psychosocial support. Subtype 5, Similar to subtype 4, but psychosocial support lasted longer than 6 months.

## Results

### Sources

We describe the selection of sources for evidence in the flow diagram shown in Figure 1.

This review included 22 studies, with 17 (Whipple, 2000; Brisch et al., 2003; Melnyk et al., 2006, 2008; Olafsen et al., 2008; van der Pal et al., 2008; Newnham et al., 2009; Meijssen et al., 2010, 2011; Nordhov et al., 2010; Ravn et al., 2012; Borghini et al., 2014; Hoffenkamp et al., 2015b; Castel et al., 2016; Evans et al., 2017; Beebe et al., 2018; Petteys and Adoumie, 2018) identified from PsycINFO and/or PubMed and 5 (Browne and Talmi, 2005; Kaaresen et al., 2006; Schroeder and Pridham, 2006; Glazebrook et al., 2007; Cho et al., 2013) identified through citation search in relevant articles. Two of the interventions were trialed with two different studies and parent–infant relationship outcomes (Melnyk et al., 2006, 2008; Meijssen et al., 2010, 2011), and one intervention was trialed three times (Kaaresen et al., 2006; Olafsen et al., 2008; Nordhov et al., 2010). Thus, there were 18 different early psychosocial parent–infant interventions but 22 studies with parent–infant relationship outcomes (Table 2). Out of all 22 studies, 19 (86.4%) were randomized-controlled studies and 3 (13.6%) were case–control studies. All included studies were quantitative with gathering and analysis of qualitative data. In total, 1,964 infants with their mothers and in some cases with their fathers were included in the final analyses (both the study and control). There were 128/1,964 term-born infants as controls, and the rest of the children were preterm-born infants (study and control). The inclusion and exclusion criteria of the studies, the participation of mothers and fathers, the content of the interventions, and outcomes related to the parent–infant relationship were themes that were studied and are presented in Table 2.

**Table 2:** Early psychosocial parent–infant interventions and parent–infant relationship outcomes.

Author, parent–infant pair (indicated separately (*) if father also participated), number of infants in final analysis, inclusion and exclusion criteria, country	Name, background theory, content, and provider	Study design, parent–infant relationship outcome measures and results (mentioned separately if included both parents)
Whipple (2000) Parent–infant pairs* 20 (intervention: 10, passive control: 10). GA ≤ 37 weeks, birthweight ≤2,500 grams, clinically stable and referred to this intervention with the mother’s consent. United States	*No name.* Based on music therapy and earlier studies on how environmental factors, physiological responses, and stress influence the development of the infant and how parents can relieve the infant’s state through learning to read the infant’s cues and by appropriate stimuli. Both groups received education sessions in the NICU. The intervention group also had individual parent training through music therapy up to one month after discharge. The first session (15–30 min) was for music and multimodal stimulation, including massage techniques, education of signs of an infant’s overstimulation, and techniques to avoid overstimulation. The second session (30 min) included education on tactile stimulation and the soothing of the infant. If the parent was insecure, the provider arranged a third session if needed (*n* = 4 parents). Provider not clearly indicated.	Case–control study After two sessions of observation (*Pre- and Post-Stress Level, Appropriate Parent Score,* both parents) and questionnaires (*Parent–Neonate Interaction Survey*). In the intervention group, the stress level of infants decreased, parents’ appropriate use of music in interaction increased, and parents spent more time with their infants than in the control group (*p* < 0.05). There was no difference at follow-up about one month after discharge.
Browne and Talmi (2005) Parent–infant pairs 84 (intervention: 28 + 31, active control: 25). GA ≤ 36 weeks, without congenital anomaly or need for surgery, expected length of stay ≥2 weeks. Mother >17 years, documented presence with infants in NICU, no major medical complications in delivery, English speaking and reading ability. United States	*No name.* Based on parent–infant interaction and its regulative meaning for preterm infants and how to support parents in finding and responding to an infant’s cues. One (45 min) session in NICU; two intervention groups and one group as control: Group 1 Demonstration and Interaction: Demonstration of the infant’s reflexes, attention/interaction, motor capabilities, and sleep/wake states by systematic Assessment of Preterm Infant Behavior protocol. Group 2 Education: Only electronic or written educational material. Content: The strengths and skills of preterm infants (stress, consoling an unhappy baby, bringing a baby to an alert state, sleep, and self-comforting skills) and the typical thoughts and feelings of the parent. Group 3 Controls: A 30–45-min discussion regarding follow-up care for preterm infants. Experimenter.	Randomized-controlled study (RCT) One month after discharge, questionnaires (*Knowledge of Preterm Infant Behavior, Parenting Stress Index*) and video observation on interactions during feeding (*Nursing Child Assessment of Feeding Scale*). Knowledge was highest in Group 2 and was higher in Group 1 than in the controls (*p* < 0.001). Interaction during feeding was best in Group 1, then in Group 2, and finally in controls (p < 0.05). Control mothers expressed the greatest stress in accepting the infant. Morbidity of the infant and the age of the mother are associated with outcomes.
Schroeder and Pridham (2006) Parent–infant pairs 16 (intervention: 8, active control: 8) GA ≤ 28 weeks, appropriate for GA in weight. Mothers ≥18 years, English speaking. United States	*Guided participation (GP).* Based on earlier studies on parents’ representations of the infant and the influence on the parent–infant relationship. GP aims to support the development of mothers to be more attuned and with adaptive expectations and intentions. Six education sessions from 30 weeks of GA (45 min, weekly in NICU), separate protocols for intervention and control groups, controls got more general information regarding prematurity and mothers in intervention group got more individual support on how to care for her own preterm infant to support their parent–infant relationship. The provider videotaped two mother–infant caregiving interactions, where the mother was holding the infant at 32 weeks and feeding the infant at 35 weeks of GA. Research nurse.	RCT At baseline (at 29 weeks of GA) interview and at follow-up video assisted interview (at 32–33 and 35–36 weeks of GA) and questionnaire (*Internal Working Model of Relating to the Baby*, *Relationship Competencies Assessment*). Mothers in the intervention group were more attuned and adaptive to their infants’ needs than mothers in the control group (*p* < 0.02).
Melnyk et al. (2006) and Melnyk et al. (2008) Parent–infant pairs* 251 (intervention: 138, active control: 113). GA 26–34 weeks, birthweight <2,500 grams (appropriate for GA in weight), anticipated survival, singleton, no severe handicapping condition. Mother and father ≥18 years, no other infant in NICU, English speaking. United States	*Creating Opportunities for Parent Empowerment (COPE).* Based on theories of self-regulation and control of infants and parenting of critically ill infants. COPE is an educational-behavioral program that provides information on the appearance and behavioral characteristics of preterm infants and how parents can participate in their infants’ care. Four phases including audiotapes and written material with educational information and behavioral activities. Phase I started at 2–4 days after admission to NICU (info and tasks to note special characteristics and milestones of their infant), Phase II after 2–4 days after phase I with information and advice to identify cues of their infant of stress, interaction readiness, Phase III 1–4 days before discharge and included information about infant, interaction, and how to smooth transition from hospital to home, and Phase IV at home one week after discharge with material on how to foster a positive parent–infant relationship and the development of the preterm child. The control group received at same time general information, such as audiotaped and written information about hospital services and discharge. Research nurse delivered recorded audio-visual educational and written material (workbook).	RCT Questionnaires at baseline (*Parental Stressor Scale-Neonatal Intensive Care, Parental Belief Scale-NICU*), and in the follow-up, where the latter was repeated 1–4 days before discharge to both parents, and observation 3–6 days after baseline (*The Index of Parental Behavior in NICU*). The observer found more positive interaction, mothers reported less stress, and both parents had more positive beliefs about infants in the intervention compared to the control group (*p* < 0.05).
Glazebrook et al. (2007) Parent–infant pairs 232 (intervention: 112, passive control: 121) GA < 32 weeks without life-threatening morbidity. United Kingdom	*Parent Baby Interaction Program (PBIP)*. Based on studies about how preterm birth influences parents and about parental stress and sensitivity in the parent–infant relationship. PBIP aims to enhance parents’ observations of their baby and sensitivity to an infant’s cues. Activities are tactile, verbal, and observational. Weekly sessions from the first week after birth could continue six weeks after discharge, but the number of sessions varied (mean eight at hospital and two at home). Controls got treatment as usual. Trained research (neonatal) nurses.	RCT At three months of age, the questionnaire (*Parenting Stress Index*) and observation of maternal interaction (*Nursing Child Assessment Teaching Scale, Home Observation for Measurement of the Environment*). There was no difference between the groups, and there was no statistical comparison.
Ravn et al. (2012) Parent–infant pairs 106 (intervention: 56, passive control: 50). GA 30–36 weeks and if length of hospital stay was expected to be >8 days, parents spoke Norwegian and had no history of drug/alcohol abuse or severe psychiatric disorders. Exclusion: congenital anomalies, neurological sequelae, hearing loss, or chromosomal disorders. Norway.	*Mother Infant Transaction Program (MITP)*. Based on earlier studies on prematurity and its effect on the sensitivity of parents and infants. MITP aims to help parents, through instruction, observation, modeling, and education, to appreciate their infant’s unique characteristics and make the parents more sensitive and responsive to their infants’ physiological and social cues, particularly those that signal stimulus overload. Seven one-hour sessions daily 7–10 days before discharge, which included sessions to be acquainted with the child, recognition of infant’s cues, and how to care for the infant, and four home visits during first three months (play, temperament). Controls got standard care (not specified). Neonatal nurses trained and supervised by a psychologist specialist.	RCT Administered *Parenting Stress Index*, short version, *Infant Behavior Questionnaire* at 6 months and *Parenting Stress Index* and long version of *Infant Behavior Questionnaire* at 12 months. Breastfeeding was observed at 9 months. There was no difference in experienced stress between the two groups at 6 and 12 months (*p* < 0.05). Mothers experienced less positive behavior in their infants at 6 and 12 months in the intervention group compared to the controls (*p* < 0.05). Intervention associated with longer breastfeeding (*p* < 0.05).
Kaaresen et al. (2006), Olafsen et al. (2008), and Nordhov et al. (2010) Parent–infant pairs* 214 (intervention: 71, passive preterm controls: 69, passive term controls: 74). Birthweight <2000 grams. Exclusion: non-survivors, triplets, Down syndrome, or if parents did not speak Norwegian. Norway.	*Early Intervention* (*EI*) is a modified version of MITP, based on the transactional model of development, which considers a disturbed pattern of parent–infant interaction to be a major influence on the child’s development. The aim of EI is to educate parents to appreciate and recognize their infant’s unique characteristics, temperament, and developmental potential and to promote dyadic reciprocity by modeling skills, providing verbal instruction and direct observation, offering emotional support when appropriate, and reinforcing the mother’s own initiative. Seven one-hour intervention sessions daily before discharge and four home intervention sessions at 3, 14, 30, and 90 days after discharge. Content as in Ravn et al. (2012) but EI also offered an initial session to ventilate and express negative feelings, and both mothers and fathers were enrolled. Controls had treatment as usual. A trained nurse supervised by a coordinating nurse and a senior child psychologist.	RCT Questionnaires: At 6 months (*Parenting Stress Index^1^, Infant Behavior Questionnaire^2^, Child-Rearing Practices Report^3^* for mothers), 12 months (*Parenting Stress Index^1^* for both parents*, Infant Behavior Questionnaire^2^, Child-Rearing Practices Report^3^* for both parents), and 24 and 36 months (*Child-Rearing Practices Report^3^* for both parents). Fathers and mothers were less stressed in the intervention versus control groups (*p* < 0.05).^1^ Maternal stress was negatively associated with a child’s negative temperament at six months in the intervention group but not for controls or at 12 months (*p* < 0.05).^2^ There were more nurturing child rearing attitudes after intervention at 12 and 24 months when compared to the preterm control group (*p* < 0.05).^3^
Newnham et al. (2009) Parent–infant pairs 68 (intervention: 35, passive control 33). GA < 37 weeks. Exclusion: if congenital abnormality, gross neurological damage, ≥ triplets, or if mother was non-English speaking or had drug dependency. Australia.	*Modification of the Mother–Infant Transaction Program (MITP)* is a program designed to enhance parent sensitivity with their own hospitalized preterm infants and to encourage them to use well-researched stimulation activities. The adapted MITP differs from these programs in that it trains mothers to combine and take responsibility for all approaches, to recognize and support individual infant needs, and to initiate positive stimulation (using well-researched activities) based on the mother’s learned appreciation of the infant’s regulatory and stimulation needs. During NICU stay, five sessions to learn to identify disorganization/stress of infant and how to apply findings in care; home intervention session at one month and follow-up at three months to recognize temperament of infant and how to parent a child. Controls got treatment as usual. Provider not indicated.	RCT At 3- and 6-months questionnaires (*Parenting Stress Index, Short Temperament Scale for Infan*t) and observation (*Synchrony Scale*) Compared to controls, mothers who participated in the intervention were more sensitive (p < 0.05) and infants attentive at three months and alert at three and six months (*p* < 0.01). Dyadic interaction was more synchronous at three months, there was more mutual attention in infants at six months, and mothers had less stress over infant (*p* < 0.01).
Van der Pal et al. (2008) Parent–infant pairs* 128 (intervention: 63, active control: 65). GA < 32 weeks, parents living in catchment area. Exclusion: major congenital anomaly or the mother being drug addicted. The Netherlands.	*The Newborn Individualized Developmental Care and Assessment Program (NIDCAP).* Based on earlier studies on the wellbeing of preterm infants and families and the synactive theory of development, parents observe infant’s behavior with the help of a provider using four subsystems (autonomic, motor, state organization, attention-interaction). It aims to support parents in being active in their infant’s care and in helping the infant’s regulation. The intervention started 48 min after birth, every 7 to 10 days. In one hour of observation, parents paid attention to the infants’ approach and avoiding behavior in four subsystems, 10 min before, during, and after caregiving. This was recorded on a sheet, and with this information, the provider summarized the infant’s approach and avoidance behavior and provided guidance for the parents on how to interact with their infants. Controls got the basic elements of developmental care (nest, positioning aids, dose not told). A developmental specialist.	RCT At 9 months (*Infant Behavior Questionnaire Revised*) and 12 months (*Parental Stress Index, short version*) *questionnaires for* both parents. There was no statistically significant difference in the perception of infant behavior characteristics and parenting stress between the groups.
Meijssen et al. (2010, 2011) Parent–infant pairs 112 (intervention: 57, passive control: 55). GA < 32 weeks and/or birthweight <1,500 grams and surviving. Exclusions: severe congenital abnormalities, mothers with illicit drug use or severe physical or mental illness, non-Dutch speaking, or participation in other studies. The Netherlands.	*Infant Behavioral Assessment and Intervention Program (IBAIP).* Based on the theory of self-regulatory competence in enhancing social interaction and learning competence. IBAIP is a post-discharge preventive intervention program for infants at risk and their parents. Its goal is to guide parents through observation to support their infants’ self-regulatory competence and social interactions. 6–8 home intervention sessions, provider used observational tool of IBAIP to see and help parents to see baby’s responses to sensory information. After sessions, parents received a report on the infant’s neurobehavioral and developmental progress, providing suggestions on how to support the infant’s explorations and self-regulatory competence by responsive parenting and environment modification. Later, parents were encouraged to gradually reduce their support. Controls got treatment as usual. Experienced pediatric physical therapists trained in IBAIP.	RCT At six months observation* (*Still Face*) and at 18 months half-structured interview (*Working Model of Child Interview*). Mothers in intervention showed less intrusive behavior than controls in still face observation (*p* = 0.04), but there were no differences in other relationship outcomes regarding mother’s representation on child and attachment.
Cho et al. (2013) Parent–infant pairs 66 (intervention: 26, active control: 40). GA < 36 weeks, hospitalized in the intensive care unit, no longer in critical condition, having no chromosomal anomalies, neurological disease, requiring no medical treatment other than internal medicine after hospital discharge, having a Japanese mother, and singleton. Japan.	*Japanese Infant Mental Health Program (JIMPH).* Based on infant mental health studies and European Early Promotion Project and on importance of early parent–infant interaction and relationship on child’s development, maternal mental health, and their relationship. JIMPH aims to promote maternal mental health, mother–infant interaction (cue and developmental guidance), social support to the mother, and stress reduction relevant to maternal parenting and child development. Six sessions until 12 months of age (one at the care unit and five after discharge). Intervention included work with interpersonal relationships, teaching how to respond to cues of infant, and interaction. Controls received treatment as usual and guidance regarding discharge of preterm infant and three post discharge intervention sessions by different helpers (nursing, public health, midwifery). JIMPH helper (nurse/public health nurse/clinical psychologist with training in JIMPH).	Case–control study At 12 months questionnaire (*Parenting Stress Index, Japanese version*) and observation week before discharge (*Nursing Child Assessment Feeding Scale*). Mothers’ interaction scale increased after intervention when compared to the control group (*p* < 0.05), but there was no difference in experienced stress.
Brisch et al. (2003) Parent–infant pairs* 68 (intervention: 32, passive control: 36) < 1,500 grams and GA ≤ 35 weeks. Exclusion: non-surviving, discharged, lacking common language or participation in other study. Germany.	*No name.* Based on different approaches from earlier studies combined with psychotherapeutic work. The aim of multimodal psychotherapeutic intervention is to improve parents’ coping and parent–infant interaction. Five supportive group therapy sessions in NICU, five individual attachment-oriented psychotherapy sessions for mother and father, one home visit session to promote parental self-compliance within 1 week after discharge, and at 3 months parental sensitivity training for infants’ cues. Controls got treatment as usual and daily talks with medical staff and nurses. Psychotherapist and a nurse from the NICU.	Case–control study At 14 months, parent–infant observations (*Strange Situation Procedure*) were conducted. There were no differences in attachment styles between the groups, but there were different amounts of neurologically impaired infants in the groups. Neurologically impaired infants benefited more from intervention for their attachment style than did controls.
Borghini et al. (2014) Parent–infant pairs 85 (intervention: 26; preterm control: 29; term control: 30). GA < 33 weeks. Exclusion: non-surviving, malformation, severe brain finding, neonatal abstinence syndrome, non-French-speaking family, or mental illness of parent. Switzerland.	*Early Intervention (EI).* Based on systematic family theory and transactional preventive intervention (the Neonatal Behavioral Assessment Scale, the Clinical Interview for Parents of High-Risk Infants, the Interaction Guidance). EI aims to improve parents’ observation, attention, and understanding of preterm characteristics and to promote parent–infant interactions. At GA of 33-week joint observation, 42-week Neonatal Behavior Assessment Scale followed by interview to allow emotion expression, and at 4 months, three videotaped free play sessions, one week apart, followed by interaction guidance. Preterm controls got information about their infant and relationship at 33 weeks of GA, and preterm and term controls participated in Neonatal Behavior Assessment Scale at 42 weeks without therapeutic guidance; these were without therapy intervention. Interaction guidance therapist (with nurse).	RCT At 4 months, observation of parent–infant interaction (*CARE index*). Intervention increased sensitivity of mother (p < 0.05) and co-operation of infant (*p* < 0.0001) when compared to pre-and post-intervention interaction. Mothers of preterm infants without interventions were more controlling than intervention and term-born control mothers (*p* < 0.002).
Hoffenkamp et al. (2015a) Parent–infant pairs* 150 (intervention: 75, passive control: 75). GA < 37 weeks (both ≥32 weeks and < 32 weeks to balance the degree of prematurity). Exclusion: poor understanding of the Dutch language and previous experience with a video-feedback intervention. The Netherlands.	Modification of *Video Interaction Guidance (VIG).* Based on earlier studies on early interaction and its association with development and parental responsiveness. This is aimed at being achieved through behaviorally focused intervention with positive feedback to facilitate bonding, attuned parental interactive behavior, and the well-being of parents, and the protocol is based on individual needs. At the first week after birth, there were three video sessions: a 15-min video during daily care routines at the first, third, and sixth postpartum days with the day after review with parents. The provider took video during daily moments, edited videos to focus on the infant’s contact initiatives and parents’ positive responses to these signals, and then viewed these micro-moments with parents, offering reflection and positive feedback. Controls got treatment as usual. VIG professionals.	RCT Questionnaire for both parents (*Postpartum Bonding, My Baby and I, Yale Inventory of Parental Thought and Actions, Parental Stress Scale; NICU*) at one week, 1-, 3-, and 6-months, and parent–infant observations at 1 and 6 days, 3- and 6-months (*National Institute of Child Health and Human Development Early Care Research Network*). When compared to controls, intervention enhanced sensitivity (mother *p* < 0.004, fathers *p* < 0.04) and less withdrawn behavior in mothers (*p* < 0.01) in the short term. Intervention also increased bonding, especially in fathers, and lasted up to six months (*p* < 0.02). However, the intervention did not diminish intrusive behavior or stress in the parents.
Castel et al. (2016) Parent–infant pairs 89 (intervention: 33, passive preterm control: 32, passive term control: 24). GA 28–36 weeks, without congenital anomaly or other disability seen early. Siblings were included. Term control infants born at the same time were identified retrospectively from the birth register. Parents >18 years, French speaking, without psychiatric history. France.	*Triadic Parent–infant Relationship Therapy (TRT).* Based on earlier studies on prematurity; parents’ wellbeing, stress, and triadic relations; attachment theory; and child’s development. TRT aims to improve triadic relationships during the first 18 months by alleviating parents’ stress, supporting their confidence, and expressing emotions. Twenty-two sessions with emotional sharing, supporting triadic relations and mental health of parents, and promoting understanding of infant and its development: twice per month for the first four months, then follow-up once a month on ward for up to 18 months. Controls got treatment as usual. A clinical psychologist.	RCT For both parents at discharge, a 3-, 9-, and 18-month questionnaire (*Parenting Stress Index, short*). Both mothers and fathers in the intervention group reported less stress at 18 months than at the beginning of the intervention and parents in the control group (*p* < 0.05).
Evans et al. (2017) Parent–infant pairs 120 (intervention: 61, passive control 59). GA < 32 weeks. Exclusion: non-surviving, major congenital anomalies, parents unwilling to participate at 24 months, non-English speaking mother, or infant was transferred to foster care. Australia.	*BabyTriple P (BTP)* is a modification of the Triple P − Positive Parenting Program. Based on earlier studies on the importance of infant’s environments for development and social learning theory, which include education of preterm infant’s characteristics and teaching strategies of parenting. The aim of the program is to strengthen parents’ knowledge, confidence, and skills. Four lesson sessions during NICU (if transferred to other hospital, by video) with content including survival skills, partner support, positive parenting, and responding to your baby. Continuing until post discharge. Four phone calls to help put theory in practice, tip sheets were mailed every 3 months, and text messages were sent until 12 months. Controls got treatment as usual. Facilitator with completed BTP training.	RCT At six weeks and 12 months, parent–infant interaction observation (*Emotional Availability Scales*) and questionnaire (*Maternal Postnatal Attachment Scale*). There were no differences in the interaction variables. No difference in attachment was reported at six weeks, but at 12 months the mothers in the intervention group reported a higher attachment than the controls (*p* < 0.02).
Beebe et al. (2018) Parent–infant pairs 71 (intervention: 39, passive control: 32). GA 26–34 weeks, mother without drug abuse/severe mental illness, English speaking, and no single adult home. Exclusion: birth weight < third percentile for GA, congenital anomaly, and family without two adults. United States	*Family Nurture Intervention (FNI)*. Based on studies on separation, the regulatory framework, hidden regulatory sub-processes (olfaction, touch, vocal senses), and autonomic co-conditioning between mother and infant. FNI aims to overcome the negative effects of maternal deprivation on neonatal care units. It involves parents being active in nurturing and enabling emotional closeness, bonding, and autonomic co-regulation. Starts after birth and continues through neonatal care unit stay; the dose of the intervention varies according to the availability of the infant, the mother, and the family, with an average of six hours per week. Nurture specialists encouraged bonding by cloth suffused by the infant’s smell and vice versa, and by holding. Enables parents to contact their child (eye, emotional speaking and singing, touch). Controls received standard care, such as education and, if they wanted, skin-to-skin care. Nurture specialists.	RCT At four months, observation of mother and infant (coding of gaze, vocal affect, touch). There were no differences in behavior compared to the intervention and control groups in mean, but mothers touched their infants more often and infants used more vocal affects in intervention than control group (*p* < 0.001).
Petteys and Adoumie (2018) Parent–infant pairs* 55 (intervention: 28, passive control 27). GA < 35 weeks, length of hospital stay >14 days, parents agreeable to spend a minimum of one hour by infant weekly, and English-speaking. United States	*No name.* Earlier studies on mindfulness and stress reduction, need for attunement, and psychoeducation of parents in preterm characteristics. Mindfulness-based neurodevelopmental care programs include education on mindfulness techniques (focused breathing, personal awareness, and nonjudgment of themselves and infants), principles of attunement and interaction, and neurodevelopmental care training for preterm infants to recognize infant cues. One individual education session (30–60 min) with mindfulness techniques, how to be present for infant, how to wait for cues of infant in care and interaction, then call with research team at least every other week during NICU care. Controls received treatment as usual. Trained research team.	RCT Within seven days, anticipated hospital discharge questionnaires for both parents (*Parental Stressor Scale: Neonatal Intensive Care Unit, Mother-to-Infant Bonding Scale*) and parents recorded how much time they spent in care and interaction with their infant (*Parent–infant Interaction Log*). There was no difference in bonding or overall parental stress between the intervention and control groups. However, parental stress decreased during intervention in the intervention group (*p* < 0.02) but not in the control group. The Parental–Infant Log did not provide applicable information to the study because of missing information.

RCT = Randomized control trial, GA = gestational age, age corrected for prematurity until 24 months, GP = Guided participation, COPE = Creating Opportunities for Parent Empowerment, PBIP = Parent–Baby Interaction Program, MITP = Mother–Infant Transaction Program, EI = Early Intervention, NIDCPA = Newborn Individualized Developmental Care and Assessment Program, IBAIP = Infant Behavioral Assessment and Intervention Program, JIMPH = Japanese Infant Mental Health Program, VIG = Video Interaction Guidance, TRT = Triadic Parent–infant Relationship Therapy, BTP = Baby Triple P, FNI = Family Nurture Intervention. ^1,2,3^Symbols are used to mark each trial of the studied intervention. *Fathers participated in the intervention with mother. In studies by: Melnyk et al. (2006, 2008) a total of 258 mothers and 154 fathers were included, but the number of fathers in the final cohort was not reported. In Kaaresen et al. (2006), Olafsen et al. (2008), and Nordhov et al. (2010) there were 70 mothers and 61 fathers in intervention groups at 12 months, in preterm controls 67 mothers and 51 fathers, and in term controls 72 mothers and 58 fathers. In Van der Pal et al. (2008) there were nine fathers in the intervention group who completed the observation, and five in the control group. In Hoffenkamp et al. (2015a) there were 150 families (150 mothers and 144 fathers), but the exact number of families eligible for trial participation was not feasible. In Petteys and Adoumie (2018), 83.7% of parents were mothers versus 16.3% fathers. In Brisch et al. (2003) there was no exact number of fathers provided. In Glazebrook et al. (2007) it was mentioned that PBIP can involve the whole family, but the mother was the principal recipient of the intervention; there was no number provided for fathers.

### Participants

The studies defined prematurity by gestational age (GA) and/or birth weight in these local cohorts (Table 2). In most of the studies, GA was defined simply as <37 weeks (15/22, 68.2%), but five (22.7%) of these studies excluded immature infants of <26 weeks (Melnyk et al., 2006, 2008; Beebe et al., 2018), < 28 weeks (Castel et al., 2016), and < 30 weeks (Ravn et al., 2012) of GA. However, 6/22 (27.3%) of the studies included only very preterm infants (very low birth weight or very low GA) (Brisch et al., 2003; Glazebrook et al., 2007; Van der Pal et al., 2008; Meijssen et al., 2010, 2011; Evans et al., 2017). One (4.5%) study included only extremely low GA infants (≤ 29 weeks) (Schroeder and Pridham, 2006). Moreover, 19/22 (86.4%) of the studies excluded infants because of health impairment other than prematurity (listed in Table 2; Whipple, 2000; Browne and Talmi, 2005; Kaaresen et al., 2006; Melnyk et al., 2006, 2008; Schroeder and Pridham, 2006; Glazebrook et al., 2007; Olafsen et al., 2008; van der Pal et al., 2008; Newnham et al., 2009; Meijssen et al., 2010, 2011; Nordhov et al., 2010; Ravn et al., 2012; Cho et al., 2013; Borghini et al., 2014; Castel et al., 2016; Evans et al., 2017; Beebe et al., 2018). Furthermore, 14/22 (64.0%) of the studies excluded infants and mothers if there was any adversity in the mother’s psychosocial situation (Whipple, 2000; Browne and Talmi, 2005; Melnyk et al., 2006, 2008; Schroeder and Pridham, 2006; van der Pal et al., 2008; Newnham et al., 2009; Meijssen et al., 2010, 2011; Ravn et al., 2012; Borghini et al., 2014; Castel et al., 2016; Evans et al., 2017; Beebe et al., 2018). Only a few studies systematically included fathers in the interventions (Kaaresen et al., 2006; Melnyk et al., 2006, 2008; Olafsen et al., 2008; van der Pal et al., 2008; Nordhov et al., 2010; Hoffenkamp et al., 2015b; Castel et al., 2016). Additionally, all of the studies required that parents could communicate in the official language of the country where the study was performed.

### Interventions

All of the interventions provided psychosocial parent–infant intervention for mothers, and the father participated in 11/22 (50%) of the studies (Table 2). Intervention was provided only during hospital stay in eight (36.4%) of the studies (i.e., seven of the interventions, 38.9%) (Browne and Talmi, 2005; Melnyk et al., 2006, 2008; Schroeder and Pridham, 2006; van der Pal et al., 2008; Hoffenkamp et al., 2015b; Beebe et al., 2018; Petteys and Adoumie, 2018). Three (13.6%) studies implemented interventions only after discharge (i.e., 2/18 interventions, 11.1%) (Meijssen et al., 2010, 2011; Borghini et al., 2014). Interventions were provided longitudinally in 12 (54.5%) of the studies (i.e., 9/18 interventions, 50.0%) (Brisch et al., 2003; Kaaresen et al., 2006; Melnyk et al., 2006, 2008; Glazebrook et al., 2007; Olafsen et al., 2008; Newnham et al., 2009; Nordhov et al., 2010; Ravn et al., 2012; Cho et al., 2013; Castel et al., 2016; Evans et al., 2017). The number of in-person sessions in the analyzed interventions varied during hospital stay, up to 10 (13/22 studies gave exact numbers, their mean number of sessions = 3.4) and after discharge, up to 22 (15/22 studies gave exact numbers, mean = 3.1). These variations in the number of intervention sessions seem to be related not only to the intervention *per se* but also to the health of the infant or other conditions, such as hospital transfers. Only one of the interventions lasted over a year (18 months) (Castel et al., 2016).

To gain an overview, most of the interventions based their theory on preterm infants’ need for sensitive regulation by parents (12/22 (54.5%) studies; 10/18 (55.5%) interventions) (Whipple, 2000; Browne and Talmi, 2005; Melnyk et al., 2006, 2008; Glazebrook et al., 2007; van der Pal et al., 2008; Newnham et al., 2009; Meijssen et al., 2010, 2011; Ravn et al., 2012; Beebe et al., 2018; Petteys and Adoumie, 2018). The interventions also considered one or more of the following aspects in their background and planning of the intervention: parental stress (2/22 (9.0%) studies; 2/18 (11.1%) interventions) (Glazebrook et al., 2007; Petteys and Adoumie, 2018), parental representations (1/22 (2.5%); 1/18 (5.5%)) (Schroeder and Pridham, 2006), the importance of the parent–infant relationship for the development of the child (6/22 (27.3%); 4/18 (22.2%) interventions) (Kaaresen et al., 2006; Olafsen et al., 2008; Nordhov et al., 2010; Cho et al., 2013; Hoffenkamp et al., 2015b; Evans et al., 2017), the parent–infant relationship in general (2/22 (9.0%); 2/18 (11.1%)) (Cho et al., 2013; Beebe et al., 2018), and multimodal psychotherapeutic theory (3/22 (13.6%) studies; 3/18 (16.7%) interventions) (Brisch et al., 2003; Borghini et al., 2014; Castel et al., 2016). In almost all the studies (19 (86.4%) of the studies and 15 (83.3%) of the interventions), the provider of the intervention was a staff member (a nurse, a physiotherapist, or a clinical psychologist) or an individual from a research team with education in intervention. Only one intervention was delivered by a psychotherapist who worked with a nurse (Brisch et al., 2003). In two studies, the provider was not clearly stated in the manuscript (Whipple, 2000; Newnham et al., 2009).

All early psychosocial parent–infant interventions were first classified into four main types: the education type included one (4.5%) intervention that was trialed twice with different outcomes; counseling included 12 interventions (67%); emotional support included three interventions (17%), in which one was trialed three times; and two interventions were classified as psychotherapy (11.1%).

One multimodal music therapy intervention (Whipple, 2000) described the intervention but did not clearly indicate the type of provider used, and we classified the study type as unclear. One (5.6%) education intervention (Melnyk et al., 2006, 2008), which was trialed twice and had three contacts before discharge and one after discharge, was implemented with written and audiotaped material only, and was classified as subtype 1. Two of the interventions (11.1%) had variation in delivery of the intervention (two different intervention groups and in others only 67% received the full protocol, and the remaining studies used other methods, e.g., phone calls and video presentations) (Browne and Talmi, 2005; Evans et al., 2017) and were therefore classified according to the lowest subtype used in the intervention, as subtype 1. We classified five (27.8% of the interventions) counseling interventions (Schroeder and Pridham, 2006; van der Pal et al., 2008; Ravn et al., 2012; Beebe et al., 2018; Petteys and Adoumie, 2018) as subtype 2 because they included up to four personal intervention sessions with individual approaches. Subtype 3 included all methods used in subtypes 1–2, in addition to providing over five sessions, being based on a specific theory, and training providers to implement the intervention, covering four counseling interventions (22.2%) (Glazebrook et al., 2007; Newnham et al., 2009; Meijssen et al., 2010, 2011; Hoffenkamp et al., 2015b). Subtype 4 offered a possibility for longer psychosocial support than in subtype 3, but for less than 6 months; there were two emotional support interventions suitable for this subtype (11.1%) (Kaaresen et al., 2006; Olafsen et al., 2008; Nordhov et al., 2010; Borghini et al., 2014). Subtype 5 included longer psychosocial support than in subtypes 1–4; there was one emotional support and one psychotherapy intervention that fulfilled these criteria (11.1%) (Cho et al., 2013; Castel et al., 2016). One intervention contained individual psychotherapy sessions for parents and specific parent–infant care but also multilevel support and peer support, and we classified it as subtype 6 (5.6% of the interventions) (Brisch et al., 2003).

### Parent–infant relationship outcomes in relation to early psychosocial parent–infant interventions

Almost half of the studies (10/22, 46.0%) reported parent–infant relationship outcomes for both parents and child (Whipple, 2000; Kaaresen et al., 2006; Melnyk et al., 2006, 2008; Olafsen et al., 2008; van der Pal et al., 2008; Nordhov et al., 2010; Hoffenkamp et al., 2015b; Castel et al., 2016; Petteys and Adoumie, 2018), while the remaining studies reported outcomes for either mother or child only. Moreover, parent–infant relationship outcomes were studied by 30 different methods (Table 2) by observations (16/22, 76.7%) (Whipple, 2000; Brisch et al., 2003; Browne and Talmi, 2005; Melnyk et al., 2006, 2008; Schroeder and Pridham, 2006; Glazebrook et al., 2007; Newnham et al., 2009; Meijssen et al., 2010, 2011; Ravn et al., 2012; Cho et al., 2013; Borghini et al., 2014; Evans et al., 2014; Hoffenkamp et al., 2015b; Beebe et al., 2018) and/or questionnaires (19/22, 86.4%) (Whipple, 2000; Browne and Talmi, 2005; Kaaresen et al., 2006; Melnyk et al., 2006, 2008; Schroeder and Pridham, 2006; Glazebrook et al., 2007; Olafsen et al., 2008; van der Pal et al., 2008; Newnham et al., 2009; Meijssen et al., 2010, 2011; Nordhov et al., 2010; Ravn et al., 2012; Cho et al., 2013; Evans et al., 2014; Hoffenkamp et al., 2015b; Castel et al., 2016; Petteys and Adoumie, 2018). Assessments of the intervention outcomes had varying time points: during neonatal intensive care (1/22, 4.5%) (Petteys and Adoumie, 2018); straight after intervention session (2/22, 9.0%) (Whipple, 2000; Schroeder and Pridham, 2006); days to weeks after discharge (2/22, 9.0%) (Melnyk et al., 2006, 2008); 1 month after discharge (1/22, 4.5%) (Browne and Talmi, 2005); at the age of 3 months (1/22, 4.5%) (Glazebrook et al., 2007), 4 months (2/22, 9.0%) (Borghini et al., 2014; Beebe et al., 2018), 6 months (4/22, 18.2%) (Newnham et al., 2009; Meijssen et al., 2010; Ravn et al., 2012; Hoffenkamp et al., 2015b), 9 months (1/22, 4.5%) (Castel et al., 2016), 12 months (3/22, 13.6%) (van der Pal et al., 2008; Ravn et al., 2012; Evans et al., 2014), or 18 months (3/22, 13.6%) (Brisch et al., 2003; Meijssen et al., 2011; Castel et al., 2016), or 24 to 36 months of corrected age (3/22, 13.6%) (Kaaresen et al., 2006; Olafsen et al., 2008; Nordhov et al., 2010). Many of the studies (17/22, 77.3%) reported at least some positive change in the intervention compared to controls.

### The findings of parent–infant outcomes in the four main intervention types

#### Education

This type included only one intervention but was trialed twice by Melnyk et al. (2006, 2008). They reported results about this COPE intervention with audiovisual or written material on preterm infants and parenting. This RCT study with 260 families found a positive effect of the intervention on better interaction behavior between mother and father, mothers’ decreased stress concerning the infant and environment, and mothers’ and fathers’ better beliefs about infants compared to controls. The parent–infant outcomes were assessed during NICU care and about 1 week before discharge. The interaction behavior was measured using the Index of Parental Behavior in the NICU. The study did not include infants with very low gestational age (< 26 weeks) or severe handicapping conditions, or twins or multiples, and mothers had to be over 17 years.

#### Counseling

This type included 12 interventions in which one intervention was trialed twice. Nine out of 13 studies (69.2%) and 8 of 12 interventions (66.7%) classified to this type reported some positive changes in the parent–infant relationship: improved parent–infant interaction (Whipple, 2000; Browne and Talmi, 2005; Newnham et al., 2009; Meijssen et al., 2010, 2011; Hoffenkamp et al., 2015b; Beebe et al., 2018), lessened parental stress (Browne and Talmi, 2005; Newnham et al., 2009; Petteys and Adoumie, 2018), improved maternal attunement to infant and adaptability (Schroeder and Pridham, 2006), or bonding and increased time spent with the infant during hospital stay (Whipple, 2000) compared to controls and assessed before or at six months or less. In three following counseling interventions, there were both positive and unchanged parent–infant relationship outcomes. In Meijssen et al.’s (2010, 2011) studies, the mothers’ behavior improved, but their representation of attachment did not change. In a study by Hoffenkamp et al. (2015a), the intervention improved sensitive behavior and bonding but not intrusive behavior or stress. In a study by Evans et al. (2017), there were no changes in observed interaction or in attachment at 6 weeks, but attachment at 12 months was better than in controls. Of these studies, 8/13 (61.5%) (Browne and Talmi, 2005; Schroeder and Pridham, 2006; Newnham et al., 2009; Meijssen et al., 2010, 2011; Hoffenkamp et al., 2015b; Beebe et al., 2018; Petteys and Adoumie, 2018) were RCT, and one of the studies (7.1%) (Whipple, 2000) was a case–control study. The size of the study cohorts varied between *N* = 16–112 in nine studies with positive parent–infant outcomes (Whipple, 2000; Browne and Talmi, 2005; Schroeder and Pridham, 2006; Newnham et al., 2009; Meijssen et al., 2010, 2011; Hoffenkamp et al., 2015b; Beebe et al., 2018; Petteys and Adoumie, 2018). Of these nine studies, one (11.1%) excluded very preterm (Beebe et al., 2018) and six (66.7%) sick preterm infants (Whipple, 2000; Browne and Talmi, 2005; Newnham et al., 2009; Meijssen et al., 2010, 2011; Beebe et al., 2018). and two (22.2%) mothers of young age (Browne and Talmi, 2005; Schroeder and Pridham, 2006) and four (44.4%) with health problems (Newnham et al., 2009; Meijssen et al., 2010, 2011; Beebe et al., 2018). However, three of the studies included only very preterm infants (Schroeder and Pridham, 2006; Meijssen et al., 2010, 2011). Interventions included 1–6 sessions (Whipple, 2000; Browne and Talmi, 2005; Schroeder and Pridham, 2006; Newnham et al., 2009; Hoffenkamp et al., 2015b; Petteys and Adoumie, 2018) or individually varied numbers of sessions (Beebe et al., 2018). The follow-up lasted until NICU discharge (Whipple, 2000; Petteys and Adoumie, 2018) or until one month after discharge (Browne and Talmi, 2005), 36 weeks of GA (Schroeder and Pridham, 2006), four months (Beebe et al., 2018) or six months of age (Newnham et al., 2009; Meijssen et al., 2010, 2011; Hoffenkamp et al., 2015b). Parent–infant relationship evaluation was assessed by questionnaires (Whipple, 2000; Browne and Talmi, 2005; Schroeder and Pridham, 2006; Newnham et al., 2009; Petteys and Adoumie, 2018) and observations (Whipple, 2000; Browne and Talmi, 2005; Schroeder and Pridham, 2006; Newnham et al., 2009; Meijssen et al., 2010, 2011; Hoffenkamp et al., 2015b; Beebe et al., 2018).

In total, 4/13 (30.8%) of these studies (i.e., 4/12 (33.3%) interventions) found no difference in interaction, responsiveness, attachment, or parenting stress (Glazebrook et al., 2007; van der Pal et al., 2008; Ravn et al., 2012; Evans et al., 2017) or found negative results in parent–infant relationship outcomes in parenting stress, how the mother experienced the infant, and maternal attachment (Ravn et al., 2012; Evans et al., 2017). The cohorts included 106–242 parent–infant pairs. In the studies with negative or no difference, all four studies excluded sick preterm infants (Glazebrook et al., 2007; van der Pal et al., 2008; Ravn et al., 2012; Evans et al., 2017), and in 3/4 (75%) of the studies mothers with health issues were excluded (van der Pal et al., 2008; Ravn et al., 2012; Evans et al., 2017). However, three (75%) of the studies included only very preterm infants (Glazebrook et al., 2007; van der Pal et al., 2008; Evans et al., 2017). The interventions included seven (Ravn et al., 2012) or varying numbers of intervention sessions (Glazebrook et al., 2007; van der Pal et al., 2008) or had no-person but material delivery (Evans et al., 2017). The follow-up lasted until 3 months in one study (Glazebrook et al., 2007) and about 12 months of age of the child in the remaining three studies (75%) (van der Pal et al., 2008; Ravn et al., 2012; Evans et al., 2017). Outcomes were evaluated by questionnaire in all four studies (Glazebrook et al., 2007; van der Pal et al., 2008; Ravn et al., 2012; Evans et al., 2017) and by observation of the parent–infant relationship in three-quarters of the studies (Glazebrook et al., 2007; Ravn et al., 2012; Evans et al., 2017).

#### Emotional support

This type included three different interventions in which one was trialed three times. All of the studies and interventions in this group (100%) had a positive result in parent–infant relationship outcomes (in sensitivity of mother, co-operation of the infant, interaction, less stress, and attitudes toward the infant) among 4- and 24-month-old children (Kaaresen et al., 2006; Olafsen et al., 2008; Nordhov et al., 2010; Cho et al., 2013; Borghini et al., 2014). In one study, there were positive changes in observed interactions but no changes in parental stress (Cho et al., 2013). Three out of five of these studies (60%) (Kaaresen et al., 2006; Olafsen et al., 2008; Nordhov et al., 2010; Borghini et al., 2014) were RCT and one (Cho et al., 2013) was a case–control study. The size of the study cohorts varied between 66 and 214. All five studies excluded unhealthy preterm infants (Kaaresen et al., 2006; Olafsen et al., 2008; Nordhov et al., 2010; Cho et al., 2013; Borghini et al., 2014) but included all gestational ages, and 4/5 of the studies excluded mothers with young age or health problems (Kaaresen et al., 2006; Olafsen et al., 2008; Nordhov et al., 2010; Borghini et al., 2014). The number of intervention sessions varied between five and six (Cho et al., 2013; Borghini et al., 2014), and there were more sessions at home after discharge (Kaaresen et al., 2006; Olafsen et al., 2008; Nordhov et al., 2010). The follow-up period varied from 4 to 36 months of age. The parent–infant relationship was assessed by questionnaires in 4/5 of the studies (Kaaresen et al., 2006; Olafsen et al., 2008; Nordhov et al., 2010; Cho et al., 2013) and by observation in 2/5 of the studies (Cho et al., 2013; Borghini et al., 2014).

#### Psychotherapy

This type included two different interventions and separate studies. One study (50%) found a positive correlation to the parent–infant relationship (Castel et al., 2016). Parents in this study (Castel et al., 2016) reported less stress after intervention at 18 months than those in the control group. There was no other parent–infant relationship outcome measure in this study. The study included 84 parent–infant pairs; the infants were preterm (GA 28–36 weeks, excluding the most preterm infants) without congenital anomaly or other disability, and the mothers were over 18 years, without psychiatric disease. This high-frequency intervention (22 sessions) aimed to improve triadic relationships through parental stress alleviation, supporting their confidence and emotional expression and parents in the control group. In a study by Brisch et al. (2003), there were 87 parent–infant pairs; the infants were only very preterm (<1,500 grams and GA ≤ 35 weeks), surviving beyond the intervention. This multimodal psychotherapeutic intervention included group therapy, individual attachment-oriented psychotherapy sessions for mother and father, one home intervention session to promote parental self-compliance within 1 week after discharge, and, at 3 months, parental sensitivity training for infants’ cues. The mother’s attachment to the infant was evaluated at 14 months. There were no differences between controls and mothers in the intervention (Brisch et al., 2003).

## Discussion

The aim of this scoping review was to provide structured knowledge of early psychosocial parent–infant interventions that seek to promote parent–infant relationships after preterm birth. We also aimed to summarize the evidence considering parent–infant relationships and identify potential gaps in existing interventions and/or in the used outcomes. Previous reviews on psychosocial parent–infant interventions have shown heterogeneity (Benzies et al., 2013; Evans et al., 2014; Givrad et al., 2021) but have not provided answers on how the variation in psychosocial parent–infant interventions could be understood.

To analyze and impose a structure on the content of the interventions, we classified the studied interventions into four main types and six subtypes, adapting existing intervention classification models, namely the International Classification for Health Interventions (ICHI) by the World Health Organization and the Template for Intervention Description and Replication (TIDieR) checklist for better reporting of interventions. Based on the classification, the most common type of early psychosocial parent–infant intervention to influence parenting behavior was found to be counseling, followed by emotional support. The least common types were psychotherapy for parenting behavior and education to influence parental behavior. Positive intervention effects on the parent–infant relationship were reported in 16 of 22 studies, particularly in single education (Melnyk et al., 2006, 2008), in 8/13 of the counseling interventions (Whipple, 2000; Browne and Talmi, 2005; Schroeder and Pridham, 2006; Newnham et al., 2009; Meijssen et al., 2010, 2011; Hoffenkamp et al., 2015b; Beebe et al., 2018; Petteys and Adoumie, 2018), in 3/3 of the emotional support interventions in which one was trialed three times (Kaaresen et al., 2006; Olafsen et al., 2008; Nordhov et al., 2010; Cho et al., 2013; Borghini et al., 2014), and in 1/2 of the psychotherapy interventions (Castel et al., 2016). Our results are in line with earlier studies on the positive outcomes of parental interventions (Benzies et al., 2013; Evans et al., 2014).

ICHI definitions of different types of early parent–infant psychosocial interventions were applied to the practice with preterm infants, and the following examples are from analyzed and classified interventions. These were applicable to intervention targeted at preterm infants and their parents. Education interventions concentrated on educating parents of the typical characteristics in preterm infants and how parents can participate in their infants’ care. Counseling about parenting behavior provided individual observations and guidance for parents on how to see cues in their own preterm infant and how to consider cues when interacting with their child. Emotional support for parenting behavior promoted parents’ mental health and parent–infant interaction and psychotherapy for parenting behavior focused on dyadic/triadic relationships through emotional sharing, supporting relationships and the mental health of parents, and promoting understanding of the infant’s development. Psychotherapeutic elements such as individual or group sessions were offered to deal with experiences during the birth of the child or an individual’s own childhood.

### Study characteristics

This study included 22 studies with 18 different early psychosocial parent–infant interventions, in which about half were implemented longitudinally, starting on the NICU and lasting after discharge. The studies used 30 different outcome measures of the parent–infant relationship, measured mainly after hospital discharge (21/22) and up to 18 months of age.

We identified multiple gaps in studies on early psychosocial parent–infant interventions, especially those related to the populations included in the interventions. In total, 86.4% of the studies excluded parent–infant pairs because of some health impairment in the infant (Whipple, 2000; Browne and Talmi, 2005; Kaaresen et al., 2006; Melnyk et al., 2006, 2008; Schroeder and Pridham, 2006; Glazebrook et al., 2007; Olafsen et al., 2008; van der Pal et al., 2008; Newnham et al., 2009; Meijssen et al., 2010, 2011; Nordhov et al., 2010; Ravn et al., 2012; Cho et al., 2013; Borghini et al., 2014; Castel et al., 2016; Evans et al., 2017; Beebe et al., 2018) and/or health or other adverse sociological risk in the mother (64.0%) (Whipple, 2000; Browne and Talmi, 2005; Melnyk et al., 2006, 2008; Schroeder and Pridham, 2006; van der Pal et al., 2008; Newnham et al., 2009; Meijssen et al., 2010, 2011; Ravn et al., 2012; Borghini et al., 2014; Castel et al., 2016; Evans et al., 2017; Beebe et al., 2018). Thus, the effectiveness of most the intervention is not studied with the preterm infants and their parents with high or multiple risk factors. In future intervention studies these groups would require more attention, because typically multiple risk factor exposure is more harmful than singular risk exposure for child development (Evans et al., 2013; Leppänen et al., 2023). Further, all of the studies required parents to be able to communicate in the official language of the country where the study was performed, thereby likely excluding recently immigrated populations. Fathers were not evaluated for inclusion criteria as mothers, which may be one of the explanations why mothers and fathers seemed to benefit differently from the interventions.

In the reviewed interventions, there were extreme but also moderate preterm infants, which may have influenced the results. The environment (neonatal intensive care unit) where preterm infants may be for quite a long time before discharge may be a challenge for psychosocial care. A need for other treatments and the transfer of infants was reported to affect implementation in some interventions (Newnham et al., 2009; Evans et al., 2017), and these kinds of prematurity and intensive care-related factors may have resulted in the observed variations in the number of sessions implemented. In 18 individual interventions, there were 30 different parent–infant outcome measures at varying age points of the infants, which makes comparison of the results difficult. In these studies, the parent–infant outcomes were often not the primary outcomes of the studies and therefore likely to be underpowered. In the future, the power of parent-infant outcomes should be considered when designing an intervention study and at least adequately discussed in the reports. Evans et al. (2014) described the contents of early psychosocial intervention and statistically compared outcome variables between study and control groups. They found that part of the interventions, especially those with education/counseling of cues and how to interact with infants, seemed to be beneficial for parent–infant relationships. However, both interventions and outcome methods, as well as follow-up times, have varied strongly in reviewed and earlier studies, details have been missing, and numbers have been small in studies, leaving uncertainties (Benzies et al., 2013; Givrad et al., 2021). A recent systematic review by Lavallée et al. (2021) on early parenting intervention after preterm birth on parental sensitivity and parental stress before and after 6 months of age found mostly low or very low quality of evidence. They discussed that this could be explained by implementation failure, risk of bias, the small number of participants, and substantial heterogeneity (Lavallée et al., 2021), which is in line with our observations.

### The interventions

The most common type of early psychosocial parent–infant intervention in our study was counseling about parenting behavior (13/22 of the studies and 12/18 of the interventions). This kind of intervention provides therapeutic and/or supportive communication in relation to behavior concerning patterns of interaction between a parent and their child/children. Nine out of 13 (69.2%) studies of the counseling type reported some significant effects on the parent–infant relationship (improvements in parent–infant interaction bonding, less parental stress, increased time spent with the child during hospital stay compared to controls), with outcomes assessed within the first 6 months of infant age. The remaining 4 of 13 studies and of 12 interventions did not report any positive intervention effects. The studies with less positive outcome results seemed to differ in that they had larger cohorts and longer follow-ups compared to studies with positive outcomes. Two of the studies with counseling interventions excluded very preterm infants, and six included only this population. Based on the subtype categories, it appears that the moderate-dose counseling interventions (subtypes 1 to 3) had a positive but maybe short effect on the parent–infant relationship. However, among the counseling interventions, intervention components, such as the amount of training/supervision of the intervention providers or the length of the intervention, did not seem to influence the results. This is interesting but may be explained by missing information about the extent to which the participants receive the intervention as intended. Intervention studies in the future, even those using an RCT design, should monitor adherence during the intervention delivery and report it (Giovanazzi et al., 2022). In three counseling interventions (in addition to one emotional intervention) that included several different measures and age points, only some of the outcomes were positive for the intervention (Meijssen et al., 2010, 2011; Hoffenkamp et al., 2015b; Evans et al., 2017). It would be important for further studies to identify the measures that are sensitive for measuring the effect of a short counseling intervention on the parent–infant relationship. Furthermore, it is important to report negative results to avoid intervention causing harmful effects.

The next most common intervention type found was emotional support to influence parenting, caretaking, or interaction, where 5/5 and 3/3 of individual interventions and studies had a positive result in parent–infant relationship outcome (e.g., improvements in maternal sensitivity, co-operation skill of the child, interaction, less parenting stress, and attitudes toward the child) assessed between 4 and 24 months of age. Emotional support provides comfort, empathy, or motivational support to the person regarding behavior concerning patterns of interaction between a parent and their child/children. Many of the emotional support interventions included the possibility of more intense and/or longer-lasting psychosocial support than the counseling interventions did (Subtype 4). All interventions included also sessions after discharge, and also very and extreme preterm infants were included to these emotional interventions. Furthermore, all emotional support interventions included providers who received education for intervention delivery. The providers, if they were staff members, also collaborated or were supported by a specialist, such as a psychologist. NICU staff have reported that they experience the provision of emotional support to parents as a demanding task (Turner et al., 2014). Therefore, it is crucial to have a specialist with appropriate education who can collaborate on or support the delivery of parenting interventions with emotional support. Although some studies have reported less stress, one of the studies did not find an intervention effect on parenting stress (Cho et al., 2013). The outcomes were assessed between 4 and 36 months of the child’s age, utilizing mostly RCT study designs and relative sample sizes. Based on this scoping review, all interventions classified as emotional support interventions were all shown to be effective. However, we cannot conclude why these interventions were all effective. Thus, our conclusion is that there is a need for studies that compare different types of interventions rather than only studying the effect of one intervention at a time. Furthermore, the active ingrediencies of the interventions should be reported in detail and impact mechanisms of interventions studied, not only the outcomes.

Less common intervention types were education and psychotherapy, with 2/22 (9.0%) of the studies and 2/18 (7.7%) of the interventions being psychotherapy and 1/18 (5.5%) being education-based. Education aims to improve knowledge to influence behavior concerning patterns of interaction between a parent and their child/children, including the nature and degree of monitoring and supervision, involvement and engagement, discipline, nurturing, and the expression of affection. An education intervention study by Melnyk et al. (2006, 2008) delivered education materials to parents but had a positive effect on parent–infant interaction as it diminished stress and improved parents’ representations of their child/children at 1 week before discharge. Unfortunately, no later outcomes were reported. This intervention also excluded most preterm infants. One of two psychotherapy interventions by Castel et al. (2016) reported less parental stress when a child was 18 months old. There were no other outcomes assessed, although that intervention lasted until 18 months and with an educated provider and background theory of the interventions to improve triadic relationships (between mother, father, and infant). Thus, the quality of the parent–infant or triadic interaction was not evaluated as an outcome in the study. In another psychotherapeutic intervention study by Brisch et al. (2003), only the attachment quality of the child was evaluated after a very multimodal intervention that could have also eased parental stress and anxiety, and improved parental representations, which were evaluated in other studies. Although child attachment was a well-founded choice for the main outcome of this intervention with an attachment-oriented approach, the narrow choice of outcomes may leave invisible some of the important effects. This study found that effect was only present in a subgroup of preterm infants, those with neurologic delay seemed to benefit from the intervention (Brisch et al., 2003). Both psychotherapeutic interventions included only very preterm infants, although not extremely preterm infants (< 28 weeks) (Castel et al., 2016). It may be that preterm infants and their parents with comorbidities, psychotherapeutic interventions are appropriate.

We found some constraints in the reviewed studies that may affect the generalization of the results. We do not know how the exclusion of mothers and infants with additional risks has influenced the studied outcome. However, some of the different intervention types showed that some subgroups benefited more from interventions than other parents in the study. Hoffenkamp et al. (2015a) reported that mothers who were traumatized benefited more from video-based interaction guidance than those who were not. And above discussed finding by Brisch et al. (2003), that if an infant had a neurological impairment, intervention improved maternal attachment, which is line with earlier results on attachment study By Korja et al. (2012) on preterm infants and parents. Prematurity *per se* might not be a risk for attachment. Overall, future interventions should pay more attention to subgroups of preterm infants and parents who may be at even greater risk of parent–infant relationship problems than preterm infants and parents without comorbidities. Previous reviews have also recommended the identification of families at risk (Benzies et al., 2013; Evans et al., 2014; Givrad et al., 2021). Psychosocial risks are known to accumulate in families with preterm infants and influence the outcomes of preterm-born children (Schothorst et al., 2007; Nosarti et al., 2012; Leppänen et al., 2023). However, adequate parent–infant emotional interaction can be protective (Yrjölä et al., 2022). Therefore, when infants and parents with cumulative risks are included in intervention trials, interventions should be tailored to meet the different and probably targeted needs of the families.

The included studies used over 30 different outcome measures; some seemed to fit well with the content of the intervention, while others did not. Pilot studies should be used to identify the sensitive outcomes of an intervention to ensure a better choice of outcomes. Standardized outcome sets could also be co-created between researchers and parents. Only 5/18 (Kaaresen et al., 2006; Glazebrook et al., 2007; Olafsen et al., 2008; Meijssen et al., 2010, 2011; Nordhov et al., 2010; Hoffenkamp et al., 2015b) of the interventions clearly reported the parent–infant relationship outcome as the primary outcome in their study. Another identified gap in previous research is related to the lack of knowledge about the intervention’s effects on fathers. In the future, the inclusion of fathers in the intervention studies should be encouraged, and the content of the intervention should be modified based on existing knowledge about early fatherhood. Further, the intervention effect on the father–infant relationship could be analyzed separately in a review.

## Limitations

Our goal was to include various types of interventions in this scoping review; however, our inclusion criteria, as stipulated in the study methodology, necessitated the exclusion of many interventions, which might have influenced the overall picture of early parental interventions. As we aimed to scope the field broadly and to identify existing gaps, we did not follow any specific quality assessment tool for the inclusion of studies. This may have influenced our observations.

We could not extract all details from a few manuscripts for classification, even though we searched the data—for example, we searched earlier publications on the intervention—and this might have influenced how we classified the interventions. As there were no readily available classification systems, our system provides a pilot for the development of other classifications in future studies. We excluded interventions that included only skin-to-skin care to concentrate more on psychological support. Skin-to-skin care *per se* has been reported as beneficial for parents and infants (Neu and Robinson, 2010; Holditch-Davis et al., 2014). We used only PubMed and PsycINFO as data sources; extension to other databases and gray literature could have expanded the picture of what kind of early psychosocial parent–infant interventions there might be. For example, in a recent complementary search we identified new types of interventions, including online/app-based interventions for parents, but these were excluded. Generally, this study could not consider other social and health care services offered to families in each NICU, hospital district, and community that might supplement interventions but were not studied in this scoping review. In the future, there is a need to describe all levels of support in different phases and environments. This field has no established terms (i.e., types and subtypes), and we used new terms compared to earlier reviews (Benzies et al., 2013; Evans et al., 2014).

## Conclusion

This scoping review study provided systematic information on studies of early psychosocial parent–infant intervention in the context of neonatal intensive care. The included interventions were classified using the ICHI, provided by the World Health Organization, and their implementation was evaluated using the TIDieR structure for reporting interventions. The most common intervention types in the studies were consultation about parenting and emotional support for parenting. Psychotherapy and education intervention types were less common. All types of interventions were shown to be beneficial for the parent–infant relationship, particularly in populations of preterm infants, without accumulating risk factors and with short-term follow-up times. But we think that in future studies, it would be of great benefit to conduct fidelity analyses of the intervention delivery (Ibrahim and Sidani, 2016) to understand how well the intervention succeeded.

Due to the heterogeneity of the outcomes and interventions used, it is hard to make comparisons between the interventions. To obtain reliable and comparable data, standardized study protocols (e.g., time of measurement and length of follow-up) and outcome measures are needed in the future. It would be important to have a long-term follow-up to study whether early outcomes are maintained. For example, synchronicity between child and parent may be a potential stable factor to measure (Feldman, 2012). The biomarkers of parenting could also be interesting proximal outcomes of parenting interventions in NICU context (Hajal and Loo, 2021). There is also a need for more intervention studies that include subpopulations of preterm infants and their parents with accumulating risks. However, it is important to carefully consider the type of intervention that is appropriate for these subpopulations. It may be that emotional support and psychotherapeutic types of interventions involving multiprofessional collaboration are more likely to be beneficial for families with accumulation risks than education or consultation. This study may help NICU professionals understand the differences between various psychosocial interventions for parenting. It may also have implications for the development of health care services for families with preterm infants.

## Data availability statement

The original contributions presented in the study are included in the article/supplementary material, further inquiries can be directed to the corresponding author.

## Author contributions

ML: Conceptualization, Data curation, Formal analysis, Investigation, Methodology, Writing – original draft, Writing – review & editing. RK: Conceptualization, Investigation, Methodology, Supervision, Writing – original draft, Writing – review & editing. PR: Conceptualization, Methodology, Project administration, Supervision, Writing – review & editing. SA-B: Conceptualization, Investigation, Methodology, Supervision, Writing – original draft, Writing – review & editing.

## Footnotes

^1^ https://icd.who.int/dev11/l-ichi/en
